# An Unexpected Phenomenon: A Case of Citalopram-Induced Dyskinesia

**DOI:** 10.7759/cureus.61364

**Published:** 2024-05-30

**Authors:** Fabienne J Jean, Michael Poulose

**Affiliations:** 1 Neurology, Touro College of Osteopathic Medicine, New York City, USA; 2 Emergency Department, St. Joseph Medical Center, Bethpage, USA

**Keywords:** dyskinesia, neurology, citalopram, adverse drug reaction, antidepressant drug, selective serotonin receptor inhibitor (ssri), dopamine receptor blocking agent, movement disorders and tremors, ssri induced extrapyramidal symptoms

## Abstract

Dyskinetic movements are characterized as hyperkinetic, repetitive movements of the extremities, facial, and oral musculature, most associated with prolonged dopamine D2 receptor blockade. In rare instances, dyskinetic movements can be brought on by selective serotonin reuptake inhibitor (SSRI) usage via an indirect D2 blockade mechanism, mimicking the D2 blockade observed with dopamine receptor blocking agents (DRBAs), such as in first-generation antipsychotics. This mimicked D2 blockade by SSRIs is said to be due to increased tonic inhibition by serotonin on dopaminergic neurons in the dopaminergic pathways of the brain, specifically the nigrostriatal pathway. In this case report, we look at a patient with a history of cerebral palsy who developed acute dyskinetic movements after short-term citalopram usage. The objective is to bring attention to the possible extrapyramidal side effects (EPS) of SSRI usage.

## Introduction

Dyskinesia refers to involuntary hyperkinetic movements of the extremities and orofacial musculature. They are most notably medication-induced, typically due to prolonged levodopa therapy in parkinsonism, prolonged antipsychotic use, and antiemetic usage, such as metoclopramide. However, recent evidence suggests that SSRIs may be associated with the development of acute dyskinetic movements in susceptible individuals. The literature on SSRI-induced EPS is scarce. Among the reported cases of SSRI-induced EPS, dyskinesia-like states account for roughly 11.3% of known EPS side effects [1]. Due to its rarity, SSRI-induced dyskinesia can be misdiagnosed as other neurological disorders such as Huntington's disease or Wilson's disease, leading to incorrect treatment. Management typically requires discontinuing the SSRI and finding alternative therapies, which can be challenging due to individual variability in response to medication. Moreover, increased healthcare utilization is a concern since patients may need frequent monitoring and additional consultations to manage adverse effects, adding to the burden on the patient.

The nigrostriatal pathway is the dopaminergic pathway that is responsible for motor function, and defects in this pathway can lead to EPS-like acute and tardive dyskinesia (TD). One proposed mechanism is that there are serotonin receptors, specifically 5-HT2C receptors, present in both the pars compacta and pars reticulata regions of the substantia nigra. The 5-HT2C subtype confers a tonic inhibition of the nigrostriatal pathway by way of gamma-aminobutyric acid (GABA-ergic) neurons [2]. These 5-HT2C receptors are found in abundance in the substantia nigra, specifically on GABA-ergic neurons. This evidence suggests that when stimulated, these receptors activate GABA-ergic neurons, leading to an increase in the concentration of GABA in the substantia nigra and an indirect inhibition of dopaminergic neurons in the nigrostriatal pathway. It has also been shown that there are GABA-ergic interneurons present in the striatum that have 5-HT2C receptors and that dopaminergic terminals present in the striatum express GABA-B receptors [2]. Thus, when serotonin binds and activates these interneurons, GABA binds to the dopaminergic terminals, leading to the inhibition of dopaminergic neurons in the striatum as well. Both mechanisms are said to cause the tonic inhibition of dopamine via serotonin. With the nature of SSRIs being that they increase endogenous serotonin, this increase in serotonin exacerbates this normal tonic inhibition, which may lead to a mimicked dopamine blockade that would be seen with DRBAs.

## Case presentation

A 47-year-old male with a past medical history of cerebral palsy, accompanied by his caretaker, presented to the emergency department with complaints of involuntary, flailing movements of the left upper extremity, excessive, purposeless tongue protrusion, and increased agitation that had been present for the last four days. The patient’s medications included baclofen for muscle spasticity secondary to his cerebral palsy and glycopyrrolate for excessive salivation, both of which he had been taking for the last five years with no adverse effects. The patient’s primary care provider recently added the SSRI, citalopram 20 mg QD, for anxiety, and the caretaker reported that the patient had been taking the citalopram as prescribed for the last two weeks. The caretaker admitted to noticing these symptoms 18 days after the patient’s first dose of citalopram and that these movements were not typical for the patient.

On a physical exam, the patient was wheelchair-bound due to his cerebral palsy. He was seemingly uncomfortable and was moving his left arm erratically and repeatedly at rest throughout the examination. Repetitive rolling and thrusting movements of the tongue were appreciated upon tongue protrusion. Cranial nerves II-XII were grossly intact; however, the patient showed difficulty performing finger-to-nose testing with his left arm due to repeated erratic movements. The overall neurologic exam was unremarkable, except for the upper and lower limb spasticity noted on the exam. Given the clinical presentation, medication history, and absence of alternative causative factors, a diagnosis of SSRI-induced dyskinesia was established. The patient was administered oral diphenhydramine 25 mg, resulting in the resolution of symptoms. During the shared decision-making process with the patient and the caretaker, it was agreed that the patient would discontinue the citalopram and schedule a follow-up appointment with the patient’s primary care provider to discuss alternative anxiolytic therapy.

## Discussion

This case report highlights an important clinical scenario involving a middle-aged male with a history of cerebral palsy who presented with symptoms suggestive of acute dyskinesia induced by the SSRI, citalopram. The patient's clinical presentation, including the onset of symptoms shortly after initiating citalopram therapy, the absence of alternative causative factors, and the resolution of symptoms following administration of diphenhydramine, strongly supports the diagnosis of SSRI-induced dyskinesia. Notably, the patient had been stable on baclofen and glycopyrrolate for multiple years without experiencing adverse effects, underscoring the likelihood of citalopram being the culprit in this case.

Management of acute dyskinetic movements primarily involves discontinuation of the offending medication, when feasible, and symptomatic treatment to alleviate acute symptoms. Due to the acute nature of symptoms, the patient’s first encounter with these symptoms, and the emergency room setting, this patient was given oral diphenhydramine, which resulted in a complete resolution of symptoms. Tardive dyskinesia refers to persistent, irreversible dyskinetic movements lasting for at least six months. The treatment of choice is vesicular monoamine transporter-2 (VMAT2) inhibitors. The VMAT2 inhibitors valbenazine, tetrabenazine, and deutetrabenazine were approved by the Food and Drug Administration in 2017 to be used as first-line treatments for tardive dyskinesia. VMAT2 inhibitors block the transport of dopamine into presynaptic vesicles; it is then rapidly degraded by monoamine oxidase, which leads to a depletion of presynaptic dopamine and a reduction in involuntary movements [3-4]. In this case, prompt recognition of the adverse drug reaction and cessation of citalopram, coupled with the administration of diphenhydramine, led to the resolution of the patient's symptoms in the emergency department. However, it is crucial to emphasize the importance of close monitoring and follow-up to assess for any potential recurrence of symptoms or need for alternative therapeutic interventions.

Despite the valuable insights provided by this case, there are important limitations to consider. The scarcity of literature on SSRI-induced dyskinesia is a significant limitation; this makes it difficult to draw strong comparisons and conclusions. Additionally, the patient’s underlying cerebral palsy introduces potential confounding factors that complicate the clinical picture and interpretation of the symptoms. While the patient had been stable on baclofen and glycopyrrolate for many years, the interactions between these medications and citalopram need to be considered. Furthermore, the acute care setting of the emergency department may have influenced the choice of management, which could have differed greatly if it had been in a more controlled primary care clinical setting.

## Conclusions

Future research directions include cohort studies involving patients with cerebral palsy or other neurological conditions on SSRIs to establish a more robust correlation between SSRI usage and the development of dyskinetic movements. Identifying specific at-risk populations can also be a focus, leading to the development of specific anxiolytic regimens for patients with predisposing conditions requiring SSRI treatment. Investigating potential genetic predispositions to SSRI-induced dyskinesia can enhance understanding and prevention strategies. Multidisciplinary research involving neurologists, pharmacologists, and primary care providers can aid in developing comprehensive management guidelines for patients who are at risk of SSRI-induced dyskinesia. Lastly, with increased studies surrounding SSRI-induced dyskinesia, enhanced pharmacovigilance can become the mainstay in managing patients on these medications, ultimately improving patient care. In conclusion, this case emphasizes the importance of vigilance for the development of acute dyskinetic movements as a potential adverse effect of SSRI treatment, particularly in vulnerable populations such as those with preexisting neurological conditions. Prompt recognition, appropriate management, and patient-centered care are essential in optimizing outcomes and minimizing morbidity associated with medication-induced movement disorders.
